# A specific brain network for a social map in the human brain

**DOI:** 10.1038/s41598-022-05601-4

**Published:** 2022-02-02

**Authors:** Lu Zhang, Ping Chen, Matthew Schafer, Senning Zheng, Lixiang Chen, Shuai Wang, Qunjun Liang, Qing Qi, Yichen Zhang, Ruiwang Huang

**Affiliations:** 1 School of Psychology, South China Normal University, Guangzhou, 510631 People’s Republic of China; 2 Key Laboratory of Brain, Cognition and Education Sciences (South China Normal University), Ministry of Education, Center for Studies of Psychological Application, South China Normal University, Guangzhou, 510631 People’s Republic of China; 3 Guangdong Key Laboratory of Mental Health and Cognitive Science, South China Normal University, Guangzhou, 510631 People’s Republic of China; 4 Department of Neuroscience, Friedman Brain Institute, Icahn School of Medicine at Mt. Sinai, One Gustave L. Levy Place, New York, NY 10029 USA

**Keywords:** Neuroscience, Cognitive neuroscience

## Abstract

Individuals use social information to guide social interactions and to update relationships along multiple social dimensions. However, it is unclear what neural basis underlies this process of abstract “social navigation”. In the current study, we recruited twenty-nine participants who performed a choose-your-own-adventure game in which they interacted with fictional characters during fMRI scanning. Using a whole-brain GLM approach, we found that vectors encoding two-dimensional information about the relationships predicted BOLD responses in the hippocampus and the precuneus, replicating previous work. We also explored whether these geometric representations were related to key brain regions previously identified in physical and abstract spatial navigation studies, but we did not find involvement of the entorhinal cortex, parahippocampal gyrus or the retrosplenial cortex. Finally, we used psychophysiological interaction analysis and identified a network of regions that correlated during participants’ decisions, including the left posterior hippocampus, precuneus, dorsolateral prefrontal cortex (dlPFC), and the insula. Our findings suggest a brain network for social navigation in multiple abstract, social dimensions that includes the hippocampus, precuneus, dlPFC, and insula.

## Introduction

A fundamental feature of our social world is social interaction, or the ways in which people reciprocally respond to one another. Imagine you are about to leave your office at 5 pm, but your boss asks you to stay for another hour. What will you do? In deciding, you may take your relationship into account, such as how close you are to your boss, or how much power they wield over you. These social relationships can be thought of occurring in a two-dimensional (2D) social space of affiliation and power^1–3^. Similar to navigating in physical space^4^, social decisions may represent a kind of navigation through abstract social dimensions—so-called social navigation^3^, which relies on the formation and utilization of a cognitive map^5^.

Tolman first proposed that the cognitive map may work as an internal model of the world that allows flexible spatial decision-making^5^. O’Keefe and Nadel suggested that cognitive maps may be represented in the hippocampus (HIP), encoded by cells that track the geometry of physical space (e.g., ‘place cells’ that correlate with an animal’s current location)^6^. Subsequently, an increasing amount of evidence showed that the HIP plays a significant role in spatial representation and navigation^7–10^. More recent fMRI studies in humans have found that the hippocampal formation also supports navigation in non-physical spaces, such as perceptual, conceptual and even social spaces^3,11–19^.

Although increasing evidence^2,11–13^ shows that navigating abstract dimensions shares similar neural substrates as spatial navigation, the neural mechanism of abstract social navigation is less understood. In the current study, we sought to identify a possible brain network that quantifies social navigation. Central to this is the hippocampal system, which is a prime candidate to encode such information in a spatial format^3,10,19^. Other regions may also contribute, such as the entorhinal cortex (EC), dorsolateral prefrontal, medial prefrontal and posterior cingulate cortices^3,20–25^.

**Figure 1:**
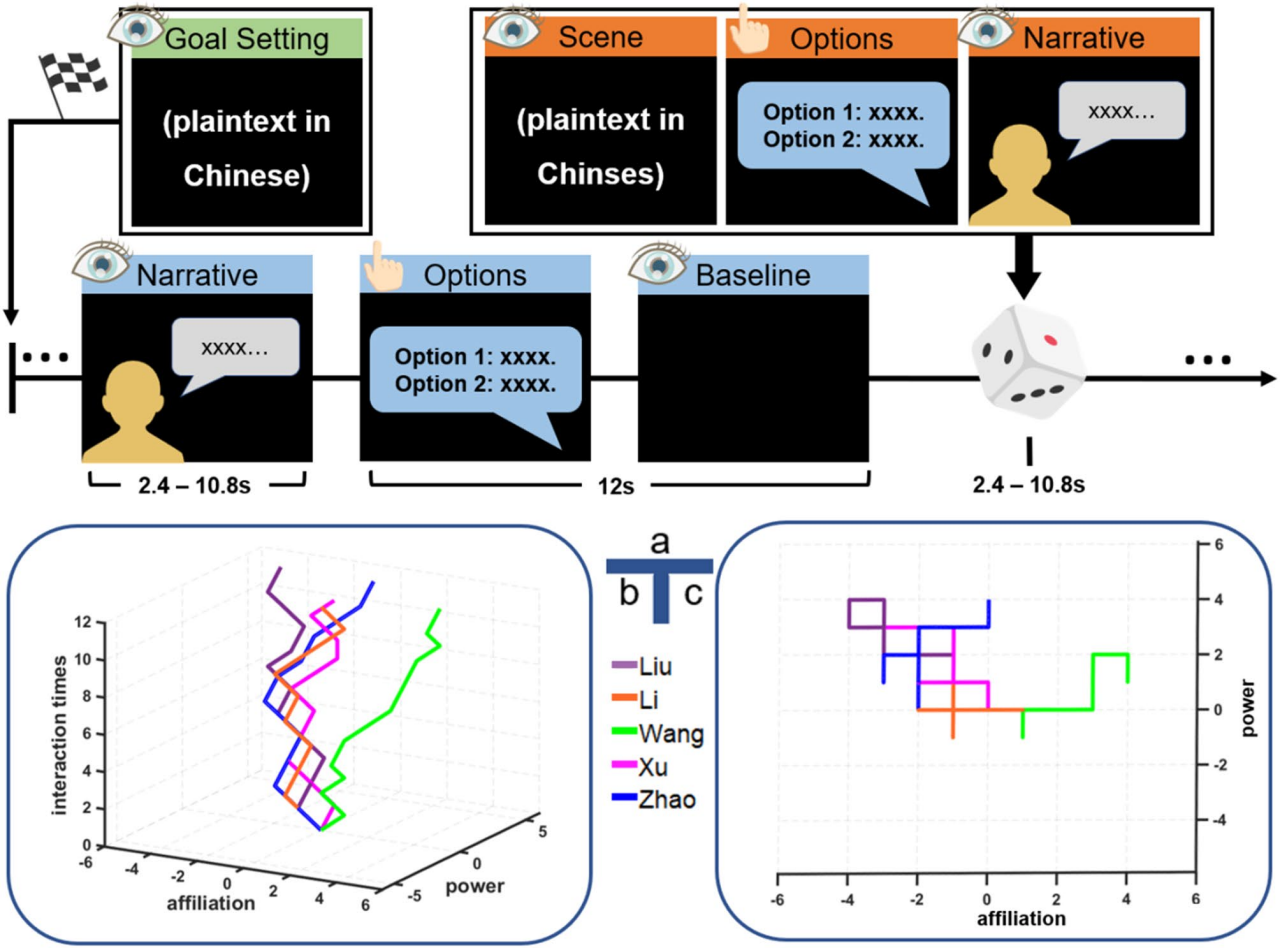
Experimental design. (**a**) Illustration of the experimental procedures. In the narrative trials, the participants watched slides that provided background information about the story or the words of a fictitious character in a grey bubble. In the option trials, the participant made a decision in a blue bubble followed by a black screen. Baseline: a black screen slide was analyzed as the baseline. (**b**) The trajectory of main characters in the 3D social space. (**c**) The trajectory of main characters in the 2D social space.

**Figure 2:**
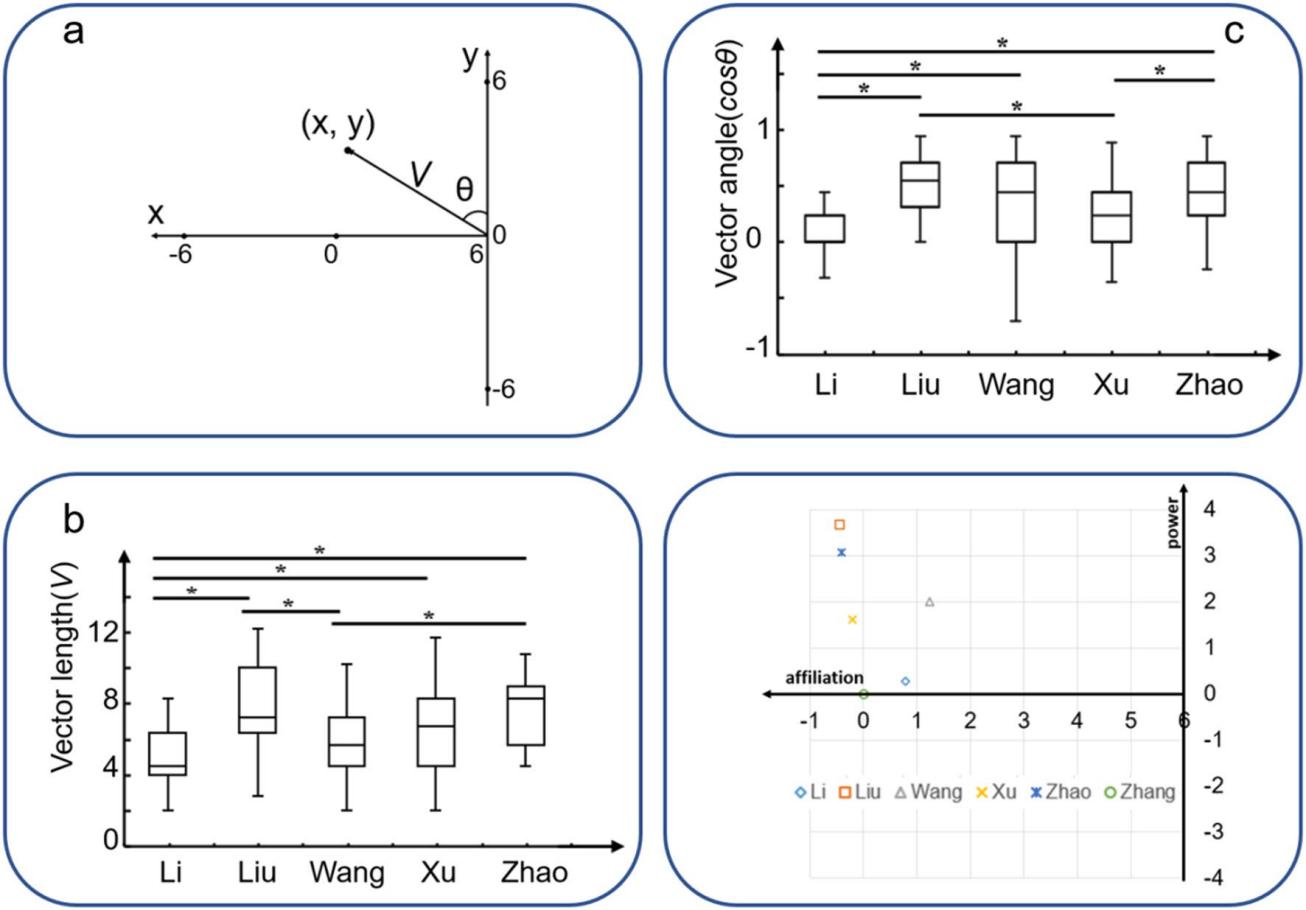
Coordinates of the characters in the story. (**a**) Schematic diagram for the 2D social map. In this 2D space, x-axis represents affiliation dimension and y-axis represents power dimension. The coordinate (*x*, *y*) represents the location of the character during the social interaction process. The participant was located at (6, 0) and the starting point for the characters was (0, 0). *V* represents the social distance between the participant and the character, and the angle represents the first-person orientation of the participant during each choice. (**b**/**c**) Plot of the length and angle for the vector *V* (*V*, *θ*). Asterisks indicate significant differences in the final locations between each pair of characters in the story. *p < 0.05. (**d**) Each symbol indicates the final coordinates of the six characters. The theoretical point of view of the participants was (6, 0), and the starting point of the characters’ origin was (0, 0).

The current study built upon the findings of Tavares et al.^2^, which showed that 2D social navigation engages the HIP during naturalistic social interactions. In a choose-your-own-adventure game (or a role-playing game) (Fig. 1), participants interacted with fictional characters in a naturalistic setting to achieve goals, such as finding a job and a place to live. Relationships between the participant and characters were modeled with a 2D geometric model of social space, framed by axes of power and affiliation (Figs. 1c and 2a). The HIP and precuneus (PCun) tracked characters’ positions in this 2D social space and additionally related to social and personality self-report variables^2^. In real life, power and affiliation have been identified as two main factors for social relations^26–31^ and two important goals that an individual could have irrespective of their demography, culture or wealth^32^. Further, quickly assessing the social status (i.e., power) and kinship (i.e., affiliation) of others is an important basis for survival and reproduction of social animals^33^.

We used this same narrative-based task to model the social relationships between the participant and fictional characters. Each character is represented as a location in 2D social space that is updated through a series of interactions in the 2D space^34^ (Fig. 2a). We quantified information about the relationships by using (1) vector length *V* (i.e., social distance) as the absolute length between the participant and the fictional characters, and (2) vector angle *cos θ*, measured as the normalized function of power modulated by affiliation, representing the first-person orientation of the participant to the character during each interaction choice. The vector angle and vector length were calculated by the following equations: $\mathit{cos}\theta =\mathrm{y}/\sqrt{{\left(6-x\right)}^{2}+{y}^{2}}$ and $V=\sqrt{{\left(6-x\right)}^{2}+{y}^{2}}$ (see “Methods” for the details).

We first aimed to replicate Tavares et al.^2^ main findings that the HIP tracks the vector angle and the PCun activity reflects the length (i.e., distance) using the same whole brain approach. Our second aim was to identify whether there is a richer neural substrate for social navigation in this task. To explore this, we first utilized an ROI approach to test the involvement of subregions in the HIP and PCun as well as other regions implicated in physical and abstract navigation, retrosplenial cortex (RSC), EC and parahippocampal gyrus (PHG)^13,35–37^. Finally, we explored the task-dependent functional connectivity during social navigation by using psychophysiological interaction (PPI) to develop a network-based model.

## Results

### Behavioral data

Twenty-nine participants (12M/17F, age = 20.3 ± 1.4 years) were included in the following analyses. Figure 1 shows experimental procedures. The task was counterbalanced for the gender of the characters; five men and eight women participated in version A, seven men and nine women participated in version B, with no significant gender difference between the two versions. Figure 1b,c show trajectories of five main characters for one participant in the 3D and 2D social space, respectively. Figure 2a shows geometric diagram of social coordinates. Significant differences were found in the egocentric vector length (*F*_4,140_ = 6.397, *p* < 0.001) (Fig. 2b) and vector angle (*F*_4,140_ = 6.157, *p* < 0.001) (Fig. 2c) between the characters. Figure 2d shows the coordinates of the five experimental characters relative to the participants at the final time point. As the neutral character, the sixth character did not change its location or coordinates (0, 0) in social space. After the fMRI scanning, each participant was also request to complete a behaviour test about assigning different size of apartments to different characters and putting all the characters in a 2D map. The results from one participant are presented in Fig. S1 (“Supplementary Materials”).

### Whole-brain parametric modulation GLM analysis: brain activation corresponding to the role-playing game

**Figure 3:**
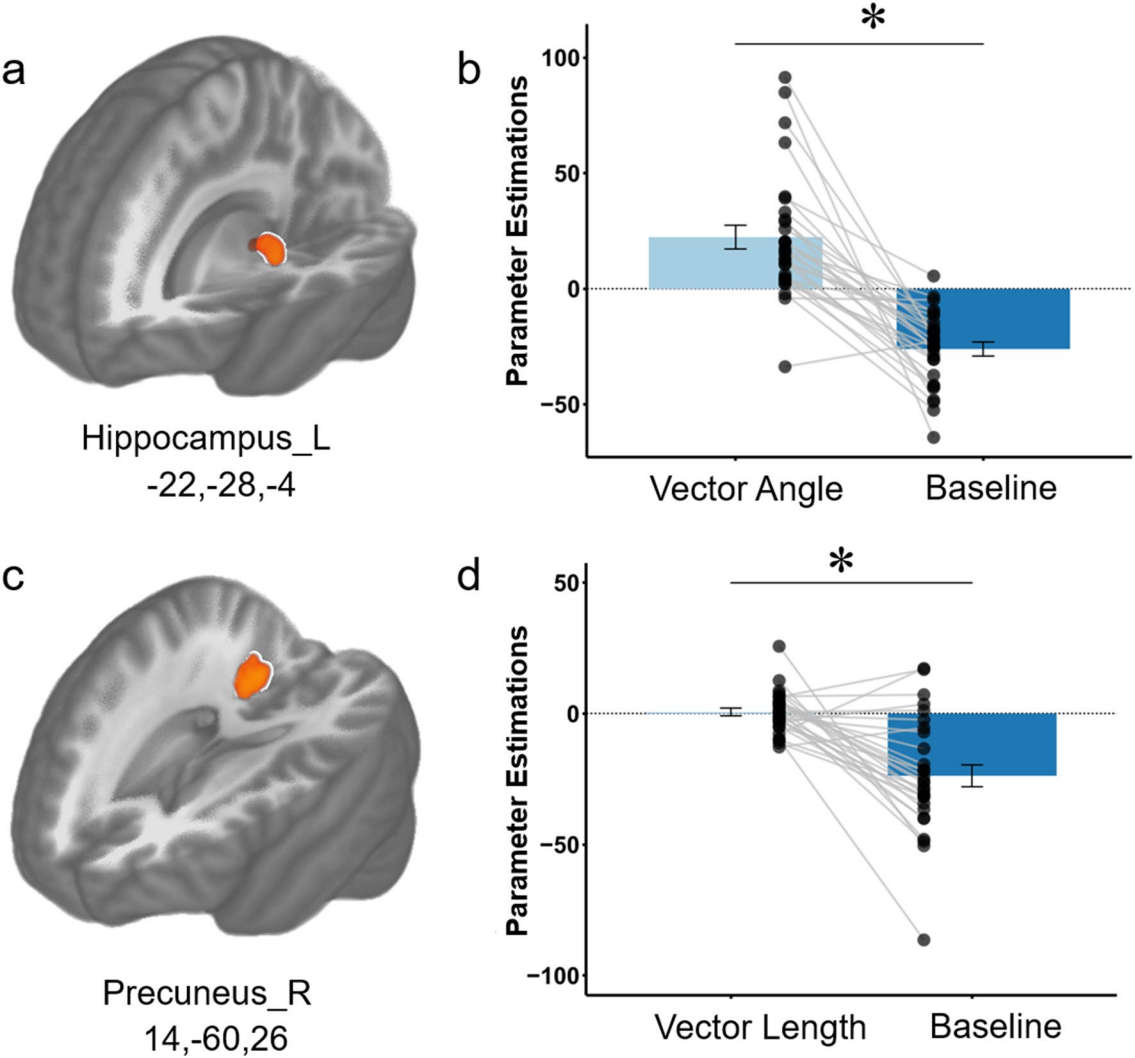
Neural correlates of power modulated by affiliation. (**a**) Brain clusters showing significant differences in activation in the hippocampus corresponding to the vector angle versus baseline contrast. (**b**) The parametric estimates extracted from the peak voxel in the left hippocampus are shown for each condition. Each dot denotes the value of parameter estimate for each participant, and error bar denotes the standard error across all participants. Asterisk * represents *p* < 0.05. (**c**) Brain clusters showing significant differences in activation in the precuneus corresponding to the vector length versus baseline contrast. (**d**) Same as (**b**) but for the right precuneus.

The whole brain analysis showed that the egocentric angle correlated with activity in left the HIP. Figure 3a shows the brain cluster in the left HIP (MNI: − 22, − 28, − 4) with significant activations for the vector angle-vs-baseline contrast. Figure 3b shows that there was a significant difference between the parameter estimates for the vector angle and baseline extracted from the peak voxel in the left HIP. The other multiple comparisons correction surviving clusters were in the right inferior occipital cortex, right thalamus, right superior temporal gyrus, and the left fusiform (see Fig. S2 in “Supplementary Materials”). Detailed information about these clusters is listed in Table 1.

**Table 1:** Brain clusters showing significant difference in activation corresponding to the vector angle versus baseline and vector length versus baseline contrasts.

Location of the peak voxel	Brodmann’s area (BA)	Peak MNI coordinate			Cluster size (# voxels)	*t-*value
		*x*	*y*	*z*		
**Vector angle (cos θ) > baseline**						
Occipital_Inf_R	BA37_R	38	− 64	− 12	4, 399	12.08
Thalamus_R		20	− 28	− 2	180	11.24
Temporal_Sup_R	BA41_R	48	− 40	16	144	6.30
Fusiform_L	BA19_L	− 28	− 72	− 12	3, 339	14.71
Hippocampus_L		− 22	− 28	− 4	141	8.84
**Vector length (V) > baseline**						
Temporal_Mid_R	BA19_R	46	− 72	0	7, 719	13.99
Frontal_Inf_Oper_R	BA44_R	38	10	30	417	6.60
Precuneus_R	BA23_R	14	− 60	26	118	6.02
Fusiform_L	BA19_L	− 28	− 72	− 10	6, 924	16.73
Thalamus_L		− 20	− 28	− 2	875	12.75

The value was obtained using one-sample *t*-test. All clusters survived Gaussian random field (GRF) correction (voxel level p < 0.001 and cluster-level *p* < 0.05). Voxel size = 2 × 2 × 2 mm^3^.

*MNI* Montreal Neurological Institute, *Sup* superior, *Inf* inferior, *Mid* middle, *Oper* opercular, *Orb* orbital, *Ant* anterior, *L (R)* left (right) hemisphere. The same below.

The whole brain analysis also showed that the egocentric vector length between the participant and the characters during social interactions was correlated with activity in the PCun. Figure 3c shows the cluster detected in the right PCun (MNI: 14, − 60, 26) that had a significant activation for the vector length-vs-baseline contrast. Figure 3d shows that there was a significant difference between the parameter estimates for the vector length and baseline extracted from the peak voxel in the right PCun. Another four clusters that survived multiple comparisons correction were in the right middle temporal gyrus, opercular part of the right inferior frontal gyrus, left fusiform, left thalamus (see Fig. S2 in “Supplementary Materials”). Detailed information about these clusters is listed in Table 1.

Table 2 showed the results for the following contrasts, power-vs-baseline, affiliation-vs-baseline, and power-vs-affiliation. We detected six clusters that corresponded to the power-vs-baseline contrast and seven that corresponded to the affiliation-vs-baseline contrast. For the power-vs-affiliation contrast, we detected ten significant clusters, nine for power > affiliation and one for affiliation > power (see Fig. S2 in “Supplementary Materials”). Detailed information about these clusters is listed in Table 2. Other results from GLM analysis are presented in Tables S1, S2 and Fig. S2.

**Table 2:** Brain clusters showing significant differences in activation corresponding to the power versus baseline, affiliation versus baseline, power versus affiliation, and affiliation versus power contrasts.

Location of the peak voxel	Brodmann’s area (BA)	Peak MNI coordinate			Cluster size (# voxels)	*t-*value
		*x*	*y*	*z*		
**Power > baseline**						
Brain stem	BA27_R	6	− 30	− 4	461	7.61
Frontal_Inf_Oper_R	BA44_R	36	6	36	266	5.82
Temporal_Mid_R	BA21_R	50	− 22	− 6	137	6.21
Cerebelum_10_R		24	− 38	− 44	113	6.38
Cingulum_Ant_R	BA25_R	8	24	− 2	106	5.43
Fusiform_L	BA19_L	− 28	− 74	− 14	17, 716	13.58
**Affiliation > baseline**						
Precentral_R	BA36_R	38	4	34	200	6.05
Temporal_Mid_R	BA21_R	52	− 22	− 8	188	6.38
Temporal_Pole_Mid_R	BA21_R	50	8	− 22	110	5.14
Fusiform_L	BA37_L	− 34	− 52	− 18	9, 585	13.07
Fusiform_L	BA37_R	34	− 48	− 16	6, 643	11.34
Precuneus_L		− 10	− 52	48	613	6.00
Thalamus_L	BA27_L	− 20	− 30	0	418	7.42
**Power > affiliation**						
Parietal_Sup_R	BA7_R	20	− 60	70	526	5.12
Frontal_Sup_Orb_R	BA11_R	20	56	− 2	190	5.03
SupraMarginal_R	BA2_R	56	− 30	34	132	4.45
Cingulum_Ant_R	BA24_R	2	36	18	117	5.09
Cuneus_L		− 12	− 64	30	3, 643	6.93
Occipital_Mid_L	BA19_L	− 42	− 78	10	193	5.10
Postcentral_L	BA2_L	− 38	− 40	68	142	4.45
Cingulum_Ant_L		0	26	30	125	4.33
Caudate_L	BA25_L	− 8	16	6	102	4.73
**Affiliation > power**						
Occipital_Mid_L	BA17_L	− 24	− 100	− 2	323	5.08

### ROI analysis: HIP and PCun subregions responding to the game

In addition to the whole-brain analysis described above, we performed ROI analyses in the HIP and the PCun with their corresponding subregions (Fig. S3 in “Supplementary Materials”).

The contrast of vector angle-vs-baseline in GLM2 showed that brain activation in the left HIP was significantly correlated with vector angle and the contrast of vector length-vs-baseline in GLM3 showed that brain activation in the right PCun was significantly correlated with vector length. The peak coordinates for these two activated clusters are listed in Table 1. However, for the contrast of angle-vs-baseline, we found that the right HIP was also activated and the peak coordinate of this cluster was located in the right thalamus (Fig. S2g in “Supplementary Materials”). Additionally, for the contrast of length-vs-baseline, we observed that the bilateral HIP were also activated and included in the clusters that extended from left thalamus and right middle temporal gyrus, respectively (Fig. S2h in “Supplementary Materials”). Thus, even though the peak coordinates were laterally located in the left HIP and the right PCun, the location of the activation clusters was extended to the bilateral HIP for the both contrasts (Fig. S2 and Table 1). For the contrast of angle-vs-baseline and length-vs-baseline, the left PCun did not survive after multiple comparisons correction according to the results of GLM2 and GLM3. However, for the contrast of optional-vs-narrative in GLM1 and the contrast of affiliation-vs-baseline in GLM4, the results showed significant activation in the left PCun (Table 2 and Table S1 in “Supplementary Materials”). In addition, previous studies showed that the bilateral PCun are involved in Theory of Mind (ToM) processes and play a role in mental imagery to represent the perspective of another person^38–40^. Considering above, we used bilateral HIP and PCun in the following analysis.

An ROI-based approach was then used to explore whether subregions in the HIP and PCun show functional specialization during the task. Previous studies indicate that the bilateral posterior HIP (pHIP) may be particularly important for encoding and retrieving high resolution spatial information relative to the anterior HIP (aHIP)^41,42^. Functional specialization also has been found in different parts of PCun. For example, a posterior part is more related to episodic memory, an anterior part is more related to somato-motor processing and self-centered mental imagery strategies, and the central part is more related to cognitive associative processes^39,43^. Considering these findings, we wanted to explore whether the subregions of HIP and PCun play different roles in this social interaction task.

Our ROI results further confirmed Tavares’ results^2^ and the results we found in the whole-brain GLM analyses, suggesting that the vector angle was supported by the HIP (left: *t* = 8.20, right: *t* = 9.67, GRF corrected for *p* < 0.001 at the voxel level and *p* < 0.05 at the cluster-level). Unexpectedly, however, ROI analyses showed that the egocentric vector length might be related to both the HIP (left: *t* = 11.56, right: *t* = 8.74, GRF corrected for *p* < 0.001 at the voxel level and *p* < 0.05 at the cluster-level) and the PCun (left: *t* = 5.15, right: *t* = 5.45, GRF corrected for *p* < 0.001 at the voxel level and *p* < 0.05 at the cluster-level) (Table S3 in “Supplementary Materials”).

### Psychophysiological interactions (PPI)

**Figure 4:**
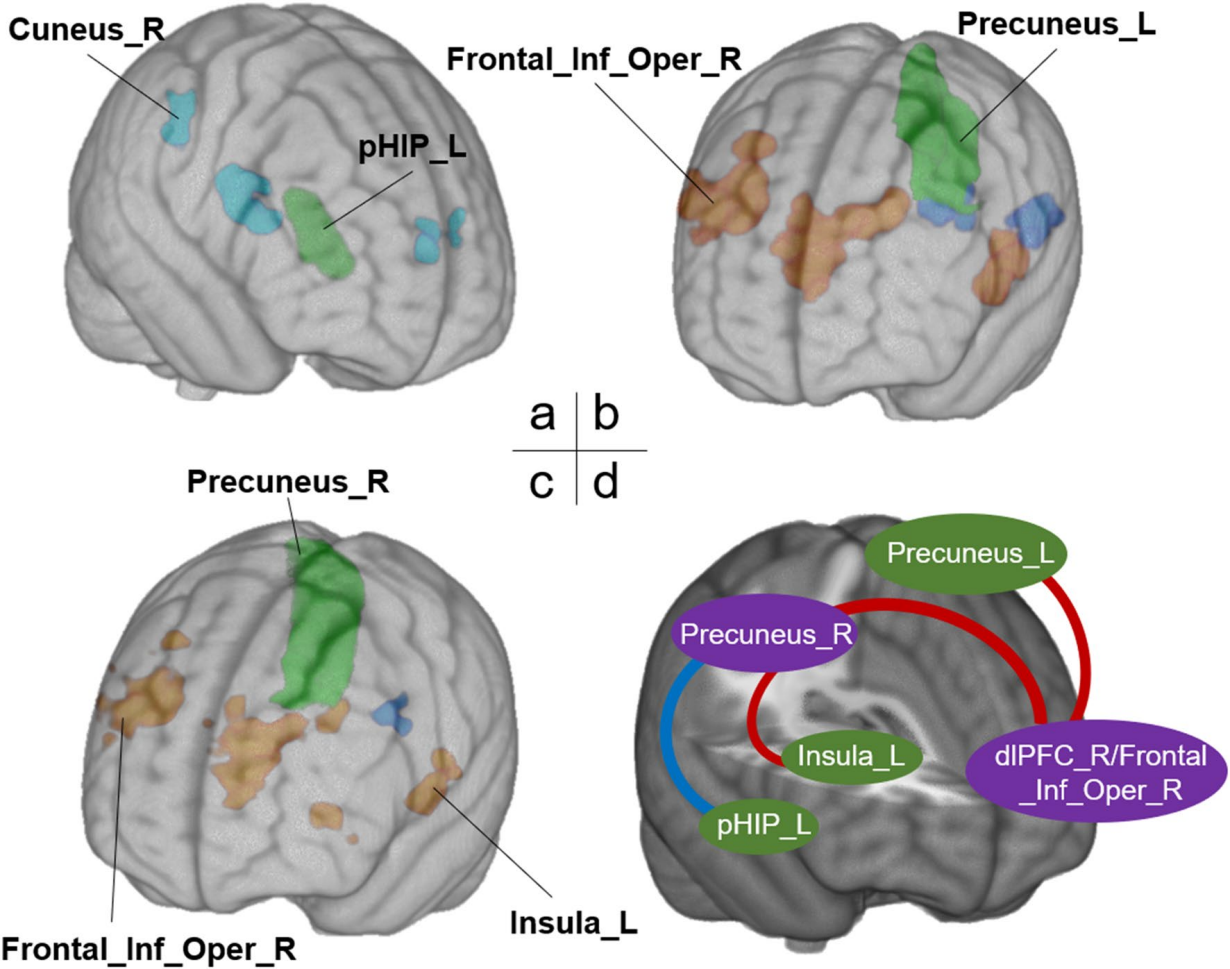
Task-dependent functional connectivity obtained from PPI analysis. (**a**) Left posterior hippocampus as the ROI; (**b**) left precuneus as the ROI; (**c**) right precuneus as the ROI. (**d**) A potential model for social navigation. Blue line indicates negative functional connectivity; Red line indicates positive functional connectivity. *pHIP* posterior hippocampus, *dlPFC* dorsolateral prefrontal cortex, *Frontal_Inf_Oper* the opercular part of the inferior frontal gyrus, *L (R)* left (right) hemisphere.

Figure S3b shows that brain activity in pHIP was significantly positively correlated with power, affiliation, egocentric vector angle and length, but no significant results survived in the aHIP. This suggests that pHIP may play a more specific role in tracking the egocentric social vector information during social navigation relative to aHIP. Brain activity in each subregion of the bilateral PCun was significantly positively correlated with power, brain activity in most of subregions (i.e., bilateral PCun_1 and PCun_2, right Pcun_3 and Pcun_4) was significantly positively correlated with affiliation, brain activity in bilateral PCun_3 was correlated with vector length, brain activity in none of subregions was correlated with vector angle. This suggests that in this social navigation task, the four subregions of PCun showed less differences between each other relative to the differences in the two subregions of HIP. Thus, we selected four ROIs, including the bilateral pHIP and bilateral PCun (instead of its subregions), to perform PPI analysis, respectively, to test a brain network-based model. Figure S4 showed flow chart of the data analysis. Figure 4 shows the results of the PPI analyses.

#### Connectivity between left pHIP and the right PCun

Figure 4a shows the results of the PPI analysis using the left pHIP as the seed ROI. We identified brain regions such as the right cuneus (MNI: 18, -70, 20; *t* = − 5.14, GRF corrected for *p* < 0.001 at the voxel level and *p* < 0.05 at the cluster-level), the left lingual gyrus (MNI: − 10, − 60, 2; *t* = -5.51, GRF corrected for *p* < 0.001 at the voxel level and *p* < 0.05 at the cluster-level), and the left Rolandic operculum (MNI: − 48, 2, 2; *t* = − 5.51, GRF corrected for *p* < 0.001 at the voxel level and *p* < 0.05 at the cluster-level) that had activity that was strongly coupled with that of the left pHIP for the optional > baseline contrast. The right PCun showed a significantly negative correlation with the activity in the seed region (the left pHIP) modulated by social decisions. The detailed information about these clusters is listed in Table 3.

**Table 3:** Task-dependent functional connectivity obtained from PPI analysis.

Location of the peak voxel	Brodmann’s area (BA)	Peak MNI coordinate			Cluster size (# voxels)	*t-*value
		*x*	*y*	*z*		
**Left posterior hippocampus as ROI**						
Cuneus_R	BA18_R	18	− 70	20	112	− 5.14
Lingual_L	BA18_L	− 10	− 60	2	315	− 5.51
Rolandic_Oper_L	BA48_L	− 48	2	2	126	− 5.51
**Right posterior hippocampus as ROI**						
Lingual_L (one-tailed)	BA19_L	− 18	− 52	− 6	104	− 4.76
**Left precuneus as ROI**						
Fusiform_R	BA37_R	38	− 58	− 14	803	7.16
Frontal_Inf_Oper_R	BA48_R	38	12	24	644	6.94
Fusiform_L	BA37_L	− 34	− 48	− 18	310	6.24
Calcarine_L	BA17_L	− 6	− 60	6	255	− 5.56
SupraMarginal_L	BA42_L	− 60	− 24	16	158	− 5.54
**Right precuneus as ROI**						
Fusiform_R	BA37_R	38	− 56	− 14	997	6.86
Frontal_Inf_Oper_R	BA48_R	38	12	24	561	6.67
Fusiform_L	BA37_L	− 34	− 56	− 14	260	6.40
Lingual_L	BA19_L	− 20	− 64	2	140	− 4.86
Insula_L	BA47_L	− 32	20	0	119	5.59

#### No significant connectivity between the right pHIP and left lingual gyrus

Table 3 shows that only one cluster in the left lingual gyrus survived after a one-tailed GRF correction. However, no cluster survived after a two-tailed GRF correction (*p* < 0.001 at the voxel level and *p* < 0.05 at the cluster-level).

#### Connectivity between the left PCun and right dlPFC

Figure 4b illustrates the results of the PPI analyses using the time-course of the left PCun as the physiological regressor. The figure shows that a positive correlation was found between the left PCun and the brain regions engaged in social interaction, such as the left fusiform (MNI: − 34, − 48, − 18) and right fusiform (MNI: 38, − 58, − 14), and the right inferior frontal gyrus (MNI: 38, 12, 24). The left calcarine fissure (MNI: − 6, − 60, 6) and the supramarginal gyrus (MNI: − 60, − 24, 16) showed significant negative task-related connectivity with the left PCun during social interaction. Detailed information about these clusters is listed in Table 3.

#### Connectivity between the right PCun and right dlPFC as well as between the right PCun and left insula modulated by social interaction

Figure 4c illustrates the results of the PPI analyses using the time-course of the right PCun as the physiological regressor. The figure shows that connectivity was found between the right PCun and the brain regions engaged in social interaction, including the left fusiform (MNI: − 34, − 56, − 14), the right fusiform (MNI: 38, − 56, − 14), the right inferior frontal gyrus (MNI: 38, 12, 24), and the insula (MNI: − 32, 20, 0). In addition, we found significantly negative task-related connectivity between the left lingual gyrus (MNI: − 20, − 64, 2) and the right PCun during social interaction. Detailed information about these clusters is listed in Table 3.

#### A potential connectivity model for social navigation

During the game, the participants watched the slides with words and were simulated the interaction with characters. This task involves language and visual processes as well as the hypothesized social navigation. In the current study, our major aim was to investigate brain regions and functional connections especially involved in the navigation of these abstract social dimensions. Thus, although several regions, such as the fusiform gyrus and the supramarginal gyrus, were suggested to be involved in not only language or visual processing but also social cognition^44–47^, we did not include these regions (such as the left rolandic operculum, supramarginal, lingual and fusiform gyri, and the calcarine fissure) in our model for a social navigation network. For example, the fusiform area has been suggested involving in the attentional processing of socially relevant cues^44,45^ and the supramarginal gyrus is also implicated in aspects of social cognition such as empathy and theory of mind^46,47^, in addition to that these regions have been reported to be associated with language and visual processes^48–51^.

#### Additional PPI analyses

By selecting the ROIs at the bilateral pHIP and PCun and taking the vector angle and vector length as covariances, we performed additional PPI analyses. The results are described as below: (1) when taking the left pHIP as the seed ROI, we observed that activity in brain regions such as the right cuneus (MNI: 18, − 70, 20), the left lingual gyrus (MNI: − 10, − 60, 2), and the left Rolandic operculum (MNI: − 48, 2, 2) was strongly coupled with the activity of the left pHIP. (2) When taking the right pHIP as the seed ROI, we did not find its significant connection with any regions in the whole brain. (3) When taking the seed ROI at the left PCun, we found not only the positive connectivity between the left PCun and three clusters, which were located in the left fusiform (MNI: − 34, − 48, − 18), right fusiform (MNI: 36, − 44, − 18), and right inferior frontal gyrus (MNI: 36, 10, 30), but also the negative connectivity between the left PCun and two clusters, which were located in the left calcarine fissure (MNI: − 6, − 60, 6) and the supramarginal gyrus (MNI: − 60, − 24, 16) during social interaction. (4) When taking the seed ROI at the right PCun, we also found the positive connectivity between the right PCun and three clusters, which were located in the left fusiform (MNI: − 32, − 56, − 14), the right fusiform (MNI: 38, − 56, − 14), and the right inferior frontal gyrus (MNI: 38, 12, 24), but also the negative connectivity between the right PCun and two clusters, which were located in the left lingual gyrus (MNI: − 20, − 64, 2) and the supramarginal gyrus (MNI: − 62, − 24, 16) during social interaction. However, the connectivity between the right PCun and left insula did not survive any more after multiple comparisons correction. The detailed information about these clusters is listed in Table S4 (“Supplementary Materials”).

## Discussion

This study analyzed social navigation in abstract social dimensions by having participants complete a choose-your-own-adventure game that simulates naturalistic social interactions in fMRI. Participants’ decisions were modeled in a 2D social space framed by power and affiliation. In whole brain analyses, we found a significant positive correlation between the HIP and the egocentric vector angle in the 2D space (Fig. 3a). We also found a significant positive correlation between the PCun and the egocentric vector length (or social distance; Fig. 3c). In PPI analyses, we found that the HIP had negative task-related functional connectivity with the PCun during these social decisions. Furthermore, positive task-related functional connectivity between the PCun and dlPFC, as well as between PCun and insula, was modulated by participants’ decisions in the social space.

Our whole-brain GLM results replicated what Tavares et al.^2^ found: left HIP BOLD activity positively correlated with egocentric (i.e., from the first-person perspective of the participant) angle (Table 1) while the right PCun correlated with the distance.

Unexpectedly, in subsequent ROI analyses we found that the HIP is not specific to the vector angle, but is also related to the vector length (Table S3 and Fig. S3b in “Supplementary Materials”). In addition, we observed a significant positive correlation between PCun activation and multiple social map indicators (power, affiliation, and the vector distances in the 2D space, Fig. S3b). Current results extend previous evidence by showing more general roles for the HIP and PCun in representing social space information.

This analysis also provided further evidence that the left pHIP in particular may play a specific role in representing egocentric vector information during social navigation (Fig. S3b). Previous studies showed that the pHIP may be particularly important for encoding and retrieving high resolution spatial information, relative to the aHIP^41,42^, suggesting the pHIP may function to encode and retrieve precise social information—such as the dynamic changes in power and affiliation from an egocentric point-of-view in a social relationship.

Increasing evidence shows that navigating along abstract dimensions may share similar neural substrates as navigation in physical space^11–13,16^. Indeed, increased hippocampal activity has been found to track both active spatial decision-making processes in physical environments^7,52^ and in our study, an active social decision-making process in a simulated social environment.

In addition, although our whole-brain GLM showed that right PCun BOLD activity was positively correlated with tracking vector length information (Table 1), a finding which is consistent with Tavares et al.’s results^2^, our results of using subregions of the PCun showed little difference between the 4 ROIs in tracking power and affiliation information during the social task (Fig. S3b in “Supplementary Materials”).

The HIP, PCun, dlPFC and insula, may be nodes in a social navigation network^3^. Previous studies provide a structural neural basis for their functional coupling: the dlPFC is connected to the HIP, the insula^53^ and the PCun^39^, and the PCun is connected to the HIP (via the RSC)^3^. In addition, the PCun contributes to mental imagery^39^ and the dynamic updating of social impressions^54^ and along with the insula may code primitive representations of relationship of the self with the outside world^39^. The insula marks salient information and transmits it to the dlPFC, suggesting it may in part guide dlPFC involvement in this task^53,55^. Increased PFC–hippocampal interaction has been found during active spatial decision-making process^56,57^. In social space, the dlPFC may work with the HIP to influence working memory expression^57^, direct attention to the locations of individuals^58^ and track social space coordinates to evaluate social relationships, ultimately facilitating social decision making^3^. To summarize, our results indicate a social navigation network, including the HIP, PCun, dlPFC and insula that may represent routes on an internal map^59^ and may play a crucial role in the deployment of social navigation.

Do the results change if the vector length and vector angle were included as covariates? To answer this question, we performed additional PPI analyses and found similar results whether we took the vector length and vector angle as covariates or not. That is, we found significant positive and negative connectivity between the seed ROIs and the brain clusters (see Table S4), except that the connectivity between the right PCun and the insula did not survive any more when the vector length and angle were included as covariates. This may suggest that the insula was involved in tracking social information about the vector angle and length in the social navigation. This result is in line with the “global emotional moment” hypothesis of the insula, which was proposed by Craig^60^ and suggested that the insula constantly receives lots of information about the location and condition of our bodies, our subjective emotions, and the key features of our environment, then incorporates important information into a “global emotional moment”. In the social navigation, the insula may receive and incorporate information about characters’ location (vector length and angle) and participants’ subjective emotions, which allow the participants to be subjectively aware of the relationship between themselves and characters at that moment, and may further affect participants’ decision making.

The current study can be built upon in several ways. First, the reduction of social navigation to 2-option decision making with a ± 1 outcome (engaging in or avoiding a private conversation or physical proximity in affiliation dimension, accepting or rejecting a demand/request in power dimension) is a simplification of real-world social interaction. Future studies could also better isolate social navigation by comparing active engagement in social interactions to passively viewing them. In addition, several regions, such as the fusiform gyrus and the supramarginal gyrus (Table 3), are reported to be involved in not only social cognition^45–47^ but also in visual or language processing^49,50^. However, it is difficult to disentangle social navigation from these cognitive processes in these regions according to present evidence. Thus, to simplify the model, we did not include these regions to the possible social navigation network. For future studies, a model that disentangle different cognitive processes in these regions might help understand the neural basis of social interactions. Further, future work should test whether the dimensions power and affiliation are orthogonal, e.g., that activity in HIP during the game is an orthogonal fusion of power and affiliation information. Our sample was comprised of undergraduates and postgraduates, which may limit the generalizability of the findings. Older participants and individuals with different kinds of social experience can be recruited for a more representative sample^61^. Another possible aim is to examine social navigation in people with psychological dysfunction—especially in disorders that feature concurrent social and hippocampal abnormalities^62^.

In summary, our results showed that the HIP and the PCun track information about social relationships in terms of egocentric angles and distances in a 2D social space of power and affiliation. Additionally, our results suggest a brain network-based model of social navigation, involving the HIP, PCun, insula, and dlPFC, that implements the computations involved in social navigation along abstract social dimensions. These results advance the understanding of the neural mechanisms underlying social navigation.

## Methods

### Participants

Forty-one healthy adult undergraduates or postgraduates were recruited from South China Normal University and nearby universities. All the participants were right-handed, with normal or corrected-to-normal vision, and no history of neurological or psychological diseases. All methods used in the current study were performed in accordance with the relevant guidelines and regulations of the ethical review board. The experimental protocol was approved by the Institutional Research Ethics Committee of the School of Psychology (Ethics number: 255), South China Normal University. All participants provided written informed consent prior to the study and were compensated for their participation.

### Experimental stimulus

The social interaction task is a role-playing game, which was designed by Tavares et al.^2^ and translated into Chinese and applied in the current study. In this game, each participant was asked to seek a job and to rent a house by interacting with six fictional characters, including five main characters and a neutral character, all with distinct personalities and social roles. The participants interacted with the characters and occasionally made decisions. A given interaction between a main character and the participant was considered either an affiliation or a power decision: “affiliation” refers to situations in which participants engage in or avoid a private conversation or physical touch, while “power” refers to situations in which participants accept or reject a request or demand. An interaction between a neutral character and the participant was neutral exchanges, such as greetings, neither affiliation nor power dimension.

Figure 1a illustrates the experimental procedures, consisting of three conditions: narrative, options, and baseline (or black condition). The role-playing game was presented to each participant by means of the slide-show and can be described as follows. (1) Narrative: the slides include background information about the story plot or the characters’ words. (2) Options: the slides indicate that participants need to make a choice. Each option slide indicated an optional trial in which the participant was requested to press the button box key-1 or key-2 to respond within 12 s to interact with the six characters. If the participant did not respond in time, the trial was considered as no response and was excluded from the data analysis. The participant needed to make 12 choices (6 in power and 6 in affiliation) related to each of the five main characters. Power reflects submissive or authoritative, and affiliation reflects the sharing of private information or physical touch. The participant also needed to make 3 choices related to the neutral character. Thus, the number of interactions and the position of each interaction in the game are fixed to ensure a fair sample of social interactions. In addition, the experimental stimuli were designed to control the total time of each character. In specific, we set the onset time of each interaction and the maximum interaction time (i.e., ≤ 12 s) for each interaction. (3) Baseline: after the participant pressed a key, the optional slide disappeared and a black slide appeared for the remaining time (12 s—reaction time in the optional trial), which was analyzed as the baseline.

The stimulus materials were originally applied in Tavares et al.^2^. In the game, the participant needs to infer or interpret the characters’ thoughts and intentions in order to make their decisions. The interpretation may be influenced by the culture of the participants. The game implemented a story about how to find a job and how to find a place to live, involving in eating in the restaurant, encountering people on the street, having interviews and so on. The scenarios for the tasks and questions are also common for young people nowadays in China. The story scripts were translated from English into Chinese by two authors (P. C. and Q. Q.) and were also rated by all of the other authors and our group members (except Matthew Schafer, an English speaker). As for the translated Chinese version, we tried our best to keep the story faithfulness and expressiveness for the game except that the characters were re-named according to Chinese culture. Thus, we are quite sure that these translated scenarios are also valid for Chinese participants.

The characters were gender-balanced, and the gender of specific characters were counterbalanced across participants. This means that the male characters in version A were switched to female characters in version B, and half of the participants received each. The experiment stimuli were prepared using PsychoPy 3.0 (http://psychopy.org).

### Characters’ location in the social space

The interactions between the participant and the characters can be represented as trajectories in a social space. The main characters changed their locations within the space when the participants made a choice on an optional trial, whereas only the neutral character never changed his/her location in the 2D social space, remaining still. Taking the *x-*axis to represent affiliation and the *y-*axis to represent power, we defined the axis unit (or scale) as follows: (1) in the affiliation dimension, when the participant engaged in or avoided a private conversation, personal touch, or physical proximity, the value on the *x-*axis changed by + 1 or − 1; (2) in the power dimension, when the participant accepted or rejected a demand/request, the value on the *y-*axis changed by + 1 or − 1. Since each participant needed to interact with each of the five main characters along each dimension (either affiliation or power), we set each axis ranging from − 6 to 6. The five main characters moved with a change in the *x-* or *y-*axis across time (*z-*axis, number of trials) in social space (Fig. 1b), whereas the neutral character’s coordinate is at (0, 0, *t*) throughout the game (*t* is interaction trial times). Non-responses were removed from the analyses as null trials.

In accordance with Tavares et al.^2^, all characters presented in the social space (Fig. 1b) were converted into a 2D plane with the *x-*axis representing the affiliation dimension and the *y-*axis representing the power dimension (Fig. 1c). In this 2D plane, the origin of the coordinates (0, 0) was the starting point of the characters: before the participant interacts with characters, each character is assumed to be neutral on both affiliation and power, neither friend nor enemy, neither superior nor subordinate. Then, with interactions and the participant making decisions, the participant re-defines the relationship between themselves and the characters. The theoretical point-of-view for the participant is at neutral power (0) and the maximum affiliation value that can be assigned to any character by the end of the task (6). Thus, characters can gain or lose power relative to the participant and can only become as affiliated with the participant as the participant’s own location on the affiliation dimension allows (6).

For each character, we calculated the vector angle *(cos θ*) and vector length (*V*) according to the coordinates of the character, in relation to the participant (i.e., egocentric, the hypothesis was supported by Tavares et al.^2^). The vector angle *cos θ* represents the normalized function of the power modulated by affiliation, and the vector length *V* represents the absolute social distance between the participant and a character. The vector angle and vector length were calculated by the following equations:

1 $$\mathit{cos}\theta =\frac{y}{\sqrt{{\left(6-x\right)}^{2}+{y}^{2}}};V=\sqrt{{\left(6-x\right)}^{2}+{y}^{2}}$$

For example, *θ* = 0 gives cos *θ* = 1 and indicates that the coordinate value of x-axis for the character is same as the coordinate value of x-axis for the participant, whose location is (6, 0). Thus, the location of the character is (6, *y*) (given *y* > 0), meaning that the participant is close to the character on the affiliation dimension and the character is higher on the power dimension.

Both the vector angle (*cos θ*) and vector length (*V*) of the final location for each character were calculated to test the differences between the different roles.

### Data acquisition

All the MRI data were acquired on a 3 T Siemens Trio MRI scanner with a 32-channel phased-array head coil. Functional images were obtained using a single-shot simultaneous multi-slice or multi-band (SMS/MB) gradient-echo EPI sequence with two dummy scans. The sequence parameters were repetition time (TR) = 1200 ms, echo time (TE) = 41.6 ms, flip angle = 52°, echo spacing = 0.81 ms, multi-band acceleration factor = 5, field of view (FOV) = 211 × 211 mm^2^, data matrix = 88 × 88, slice thickness = 2.4 mm without inter-slice gap, voxel size = (2.4 mm)^3^, anterior-to-posterior phase encoding direction (A ≫ P), and 65 interleaved slices (parallel to AC–PC plane) covering the whole brain. The field-map images were also obtained with a double echo gradient-echo sequence (TR = 735 ms, TE1/TE2 = 5.04 ms/7.50 ms, flip angle = 60°, FOV = 211 × 211 mm^2^, data matrix = 88 × 88, slice thickness = 2.4 mm without inter-slice gap and the same slice locations as those for the fMRI).

In addition, high resolution brain structural images were acquired using a T1-weighted 3D Magnetization Prepared Rapid Gradient Echo (MPRAGE) sequence with the following parameters, TR/TE = 1600 ms/2.98 ms, flip angle = 9°, slice thickness = 1 mm, FOV = 256 × 256 mm^2^, voxel size = (1.0 mm)^3^, and 176 sagittal slices.

The fMRI data were acquired when the participants were playing the role-playing game. After the scan, each participant was asked to complete a memory questionnaire to ensure he/she had attended to the task. All the MRI data for each participant were acquired in the same session.

### Preprocessing fMRI data

The fMRI data were preprocessed with FSL/FEAT (FSL 5.0.9, https://fsl.fmrib.ox.ac.uk/fsl). Before preprocessing, the T1-weighted 3D brain structural images for use in registration were brain-extracted with BET/FEAT. We stripped the skull of the brain structural images and cropped the neck, normalized the structural images into MNI standard space, and resampled to 2 × 2 × 2 mm^3^. The preprocessing steps were as follows. (1) Head movement correction: To reduce the effect of head movement, MCFLIRT was used to apply a rigid-body transformation with the middle volume as the reference volume^63^. Six mean head movement parameters (three translational and three rotational) and a summary movement index were obtained for each participant. (2) Functional images distortion-correction: *B*_0_ field-map images were used in the *B*_0_-unwarping, which was carried out with a boundary-based registration (BBR) by estimating the wrapped phase images in radians and the field-map in rad/s as well as regularizing the field-map based on the magnitude of the images. (3) Spatial smoothing: The obtained images were smoothed with a 5-mm full width at half maximum (FWHM) Gaussian kernel. (4) High-pass filtering (cut off period = 128 s) was performed to remove low frequency artifacts. Slice-timing correction was not applied to correct for the within-scan acquisition time difference between slices in that we considered the short TR in the multiband sequence. The corrected functional data were co-registered to the 3D brain structural images.

All the participants’ functional images were separately realigned and the translation and rotation correction parameters were individually examined to ensure that no participant had significant head movement. We excluded the functional datasets for 12 participants from the total 41 participants because their head movements exceeded our inclusion criteria (translation < 2 mm in any of the planes or rotation < 2° in any of the axes). The fMRI datasets of 29 participants (12M/17F, age = 20.3 ± 1.4 years old) remained for further analyses.

### Whole-brain GLM analysis: brain activation corresponding to different conditions

General linear model (GLM) is expressed as follows:

2 $$Y=X*\beta +\varepsilon ,$$

where *Y, X, β,* and *ε* are the observed data, design matrix, model parameters, and the errors, respectively. The errors *ε* is assumed a zero mean normal distribution.

GLM is widely used in fMRI studies^64,65^. The belief is that the stimulation will induce neural activity in some brain regions and induce hemodynamic response. In fMRI data analysis, GLM is usually a two-level hierarchical model, including the first-level or individual-level analysis and the second-level or group-level analysis.

In the first-level analysis, the BOLD signal time-series (*Y*) associated with each voxel is modelled as a linear combination of one or more known predictors each scaled by a parameter plus an error term in the GLM. Of interest are the columns representing manipulations or experimental conditions, although the matrix typically also includes regressors of non-interest, modelling nuisance variables such as low-frequency drifts and motion. *β* is the vector of unknown weights setting the magnitude and direction of the association between *Y* and each given predictor variable. *ε* is an *n* × 1 vector containing the error values associated with each observation (i.e., the value of each observation that is not explained by the weighted sum of predictor variables). ε is assumed zero mean Gaussian noise. Here *n* is the total numbers of TR for the task scanning.

Take GLM2 as an example, Fig. S5 (in “Supplementary Materials”) depicts the design matrix for the first-level analysis. As shown in Eq. (2), *Y* is an *n* × 1 column vector to represent the BOLD signal time-series associated with a single voxel. Here *n* is the total TR for the role-playing game. *X* is the *n* × *m* design matrix (here *m* = 14) and each column represents a different predictor variable. Figure S5 showed that left 8 columns represent the narrative condition and its temporal derivatives, optional condition and its temporal derivatives, baseline condition and its temporal derivatives, as well as the vector angle (parametric regressor) and its temporal derivatives, respectively. Right 6 columns represent the 6 head movement regressors. The aim of the first-level statistical analysis is to determine how large the contribution of each predictor variable is to the BOLD signal time-series, that is, how large each parameter is, and whether it is significantly different from zero.

For the second-level analysis or the group-level analysis, the mathematical equation as follows:

3 $$\hat{\beta } = X_{G} *\beta_{G} + \eta ,$$

where estimated parameters $\hat{\beta }$ are brought forward from the first-level analysis to the second, *X*_*G*_ is a group-level matrix specifying how the individual subjects’ data are to be related (i.e., all averaged in a single group in our study), and *η* is the error term that contains a mixture of both the fixed and random variability. In the second-level analysis, we used a one-sample *t*-test, which is the simplest possible linear model by assuming a single, homogeneous group of subjects, to test the mean response to see if it is different from zero.

In our analysis, we analyzed the fMRI data by using FEAT/FSL. For the 1st-level, we computed GLM using the time-series from the voxel level to detect brain activation corresponding to the different stimulus conditions within each participant. For the 2nd-level, we carried out a mixed-effects analysis across all the participants to obtain a group activation pattern. Four GLM analyses were carried out in the current study, and are described below.

In the first-level analysis or the single-subject analysis, the GLM can be re-expressed using Eq. (4), where *Y* represents the BOLD signal time-series associated with a single voxel. *X* is the *n* × *m* design matrix. *β* is the *m* × 1 vector of unknown weights. ε is an *n* × 1 vector containing the error values.

4 $$Y=\left[\begin{array}{c}{Y}_{1}\\ \vdots \\ {Y}_{n}\end{array}\right],X=\left[\begin{array}{ccc}\begin{array}{c}{X}_{\mathrm{1,1}}\\ \vdots \\ {X}_{n,1}\end{array}& \begin{array}{c}{X}_{\mathrm{1,2}}\\ \vdots \\ {X}_{n,2}\end{array}& \dots \begin{array}{c}{X}_{1,m}\\ \vdots \\ {X}_{n,m}\end{array}\end{array}\right],\beta =\left[\begin{array}{c}{\beta }_{1}\\ \vdots \\ {\beta }_{m}\end{array}\right],\upvarepsilon =\left[\begin{array}{c}{\varepsilon }_{1}\\ \vdots \\ {\varepsilon }_{n}\end{array}\right]$$

In specific, for GLM1 the current study, *m* = 12, the design matrix *X* includes regressors of interest *R*_A1_, *R*_A2_, and regressors of non-interest, such as motion movement regressors. For GLM2, *m* = 14, design matrix *X* includes regressors of interest *R*_B1_, *R*_B2_, and regressors of non-interest. For GLM3, *m* = 14, the design matrix *X* includes regressors of interest *R*_C1_, *R*_C2_, and regressors of non-interest. For GLM4, *m* = 16, the design matrix *X* includes regressors of interest *R*_C1_, *R*_C2_, and regressors of non-interest. Here *n* is the total number of TR for the role-playing game.

The first GLM (GLM1) contrasts the three critical conditions: we included three main regressors, narrative condition (*R*_A1_), optional condition (*R*_A2_), and baseline (*R*_A0_), which led to 3 contrasts, *R*_A2_-vs-*R*_A1,_ *R*_A2_-vs-*R*_A0_, and *R*_A1_-vs-*R*_A0_. For each participant and for each trial, the onset time of each optional trial is fixed whereas the duration time of each optional trial is different and depends on the participant’s reaction times. Thus, we included different duration time for each optional trial in the GLM regressor to fix the problem of different duration time of the optional trial. Temporal derivatives of these three main regressors and six head movement regressors were included in the model and all the regressors were convolved with the double-gamma hemodynamic response function (HRF). We calculated the brain activations corresponding to each contrast. The results were listed in Tables S1 and S2.

GLM2 was used to estimate brain activations related to the vector angle. In the first level, we constructed four different main regressors: *R*_B1_ for narrative condition; *R*_B2_ for optional condition; *R*_B0_ for baseline, and *R*_B3_ (parametric regressor) which was modulated by the parametric weight of vector angle (*cosθ*). The parametric regressor file of *R*_B3_ is a 3-column format regressor file. The 1st and 2nd columns correspond to the onset time and the duration of each optional trial, and the 3rd column is the vector angle at that trial. In this way, the parametric regressor reflect the dynamic change of characters’ location over time. We also included temporal derivatives of these four main regressors and six head movement regressors. All the regressors ware convolved with the double-gamma HRF (see Fig. S5 in “Supplementary Materials”). Then we obtained contrasts. We focused on the brain regions that showed significant activations associated with the *R*_B3_-vs-*R*_B0_ (or vector angle vs. baseline) contrast.

GLM3 estimated brain activations related to the vector length (absolute social distance). Like for the angle model (GLM2), we constructed four main regressors, narrative condition (*R*_C1_), optional condition (*R*_C2_), baseline (*R*_C0_), and one regressor modulated by the parametric weight of vector length (*R*_C3_). The parametric regressor for *R*_C3_ is similar to that for *R*_B3_, except that it estimates the weight of vector length rather than vector angle. Again, temporal derivatives of these four main regressors and six head movement regressors were included in the model and all the regressors were also convolved with the double-gamma HRF. The *R*_C3_-vs-*R*_C0_ (or vector length vs. baseline) contrast was used to determine the brain regions that showed significant activations by the vector length.

The fourth GLM (GLM4) computed brain activations related to either power or affiliation: we included five main regressors, narrative condition (*R*_D1_), optional condition (*R*_D2_), power (*R*_D3_), affiliation (*R*_D4_), and baseline (*R*_D0_). We obtained contrasts and focused on the brain activations corresponding to each of the following three contrasts, *R*_D3_-vs-*R*_C0_ (or power-vs-baseline), *R*_D4_-vs-*R*_C0_ (or affiliation-vs-baseline), and *R*_D3_-vs-*R*_D4_ (or power-vs-affiliation).

### ROI analyses in subregions of the HIP and PCun

In addition to the whole-brain analyses, we used ROI analyses to further confirm whether the HIP is related to the angle and the PCun is related to the length. This analysis is inspired by the previous study^2^, as well as increasing evidence showing that the HIP and PCun play key roles in spatial memory and navigation in both humans and animals^7,39^, while their subregions perform distinct coperations^39,41–43^. As such, we explored whether the subregions of the HIP and the PCun play different roles in the current task.

First, we parcellated the HIP and PCun into different subregions, following previous studies (Fig. S3a in “Supplementary Materials”)^13,35,41,66^ and then we analyzed their roles during the game. Specifically, we parcellated HIP into four subregions and the PCun into eight subregions across the two hemispheres.

The parcellation steps are summarized as follows. (1) We obtained the hippocampal mask in MNI space based on the AAL template. We parcellated the HIP in each hemisphere into two approximately equal parts along the long axis^13,66^. Thus, we determined the posterior HIP (pHIP) from *y* = 44 to 54 and the anterior HIP (aHIP) from *y* = 54 to 64. (2) We parcellated the PCun into four subregions, PCun-1 (dorsal-central portion), PCun-2 (dorsal-anterior portion), PCun-3 (dorsal-posterior portion) and PCun-4 (ventral portion), according to their specific anatomical and functional connectivity patterns^35^.

Second, for each of the ROI, we obtained the parameter estimates of the vector angle-vs-baseline contrast from the GLM2; the vector length-vs-baseline contrast from GLM3; and power-vs-baseline, affiliation-vs-baseline contrasts from the GLM4. A one-sample *t*-test was applied in each of the subregions for each of the four contrasts. Gender and age were used as covariates. GRF was used to correct for multiple comparisons. The threshold was set at the voxel level *p* < 0.001 and *p* < 0.05 at the cluster-level.

### Psychophysiological interactions (PPI)

Another goal was to understand the functional interactions between brain regions during the social interactions. The ROI analyses above identified four key regions. For example, the bilateral pHIP was involved in processing the vector angle while the bilateral PCun was involved in processing the vector length (see “Supplementary Materials”, Fig. S3 and Table S3), results that replicated Tavares et al.^2^. We took bilateral pHIP and bilateral PCun as seed ROIs to do PPI analyses to further our exploration.

In short, PPI analysis is a technique for measuring task-specific changes in functional connectivity between brain areas. In the current study, PPI analysis was conducted to examine the effect of social interactions on functional connectivity between a seed ROI and other regions in the whole brain^67^. An increase of task-specific inter-regional functional connectivity is usually interpreted in terms of an increased flow of information between brain areas during that task. Statistically, PPI is based on extensions to statistical models of a classic 2 × 2 factorial design, a clever use of the GLM^68^. Taking the left pHIP as an example, we have the GLM as:

5 $$Y = (optional - baseline)*\beta_{1} + (pHIP)*\beta_{2} + (optional - baseline)*(pHIP)*\beta_{3} + \varepsilon$$

To keep the above equation simple, we did not include the covariates. In this equation, the optional-vs-baseline is a psychological variable, while the activity in the left pHIP is a physiological variable. The interaction term is the effect of optional-vs-baseline and the left pHIP activity on the whole brain. The null hypothesis is that the interaction term does not contribute significantly to the model.

Again, taking the left pHIP as an example, we describe the PPI steps. The psychological variable in the model is optional-vs-baseline. The physiological variables were the brain signals extracted from the left pHIP. For each participant, we first took the left pHIP as a seed and extracted its time-course and then performed a 1st-level analysis, that is, a PPI-specific GLM analysis, by using the standard procedure implemented in FSL. In detail, we selected regressors, as follows. (1) r1: psychological regressor (PSY), a task regressor for “optional condition-vs-baseline” to model all the differences between the optional condition and the baseline. We made a 3-column format regressor including all the task trials of the optional condition and baseline. The weight, or the 3rd column value, of the optional condition was 1 and the weight of the baseline was − 1. (2) r2: physiological regressor (PHYS), the time-course of the selected seed (left pHIP). (3) r3: PPI, an interaction between r1 and r2. (4) r4: a regressor for the narrative trials. (5) r5: a task regressor for “optional condition + baseline”. Here r1, r4, and r5 were convolved with the double-gamma HRF. The parametric regressors for vector angle and length were not included as nuisance covariates in the PPI analysis. One reason is that from both the GLM and the ROI-based analyses, we found that BOLD signal magnitude in the left HIP and bilateral PCun is correlated with different social mapping variables, such as affiliation, power, vector angle and vector length. Another reason is that our aim is to find a general brain network-based model for social navigation. Subsequently, we calculated single-participant contrasts (PSY, PHYS, and PPI) to determine both the positive and negative connectivity changes between the selected seed and other brain regions in the whole-brain during social interaction.

Similarly, we carried out three other PPI analyses by taking each of the other three regions, the right pHIP and bilateral PCun, as separate seeds. The PPI models were analyzed using the steps described above, except that the average time-course, i.e., the physiological regressors, were replaced by each of the three seed ROIs.

In total, we selected four ROIs (left pHIP, right pHIP, left PCun, and right PCun) to perform the PPI analyses. In the calculations, we selected one of them as a seed ROI to estimate the task-specific changes in functional connectivity between the seed ROI and other regions in the whole brain. Thus, we set up four PPI models independently for four seed ROIs.

We also performed additional PPI analysis, in which we included parametric regressors for vector angle and length as nuisance covariates. The result is listed in Table S4 (“Supplementary Materials”).

### Statistical analysis

#### Behavioral data

A one-way analysis of variance (ANOVA) was used to test the values of the characters’ vector angles and lengths. The post-hoc threshold was set at *p* < 0.05.

#### Whole-brain GLM

We carried out a mixed-effects analysis by using a one-sample *t*-test to determine the brain regions with significant activation for the given contrast. We took gender and age as covariates. The significance level was set at *p* < 0.001 at the voxel-level and *p* < 0.05 at the cluster-level, and the GRF (Gaussian random field) approach was used to correct for multiple comparisons.

#### PPI analyses

The statistical approaches were the same as those used for the GLM analyses. In the 1st-level analysis, we added the six head movement parameters of the brain functional dataset as covariates. In the 2nd-level analysis, we took a mixed-effects analysis using a one-sample *t*-test on the single-participant beta images with gender and age as covariates. GRF approach was used to correct for multiple comparisons. The threshold level was set at *p* < 0.001 at the voxel level and *p* < 0.05 at the cluster-level.

## Supplementary Information

Supplementary Information.

## Data Availability

The data that support the findings of this study are available from OSF but restrictions apply to the availability of these data, which were used under license for the current study, and so are not publicly available. Data are however available from the authors upon reasonable request and with permission of OSF.

## Abbreviations

EC: Entorhinal cortex
alEC: Anterior-lateral entorhinal cortex
pmEC: Posterior-medial entorhinal cortex
PHG: Parahippocampal gyrus
pPHG: Posterior parahippocampal gyrus
aPHG: Anterior parahippocampal gyrus
HIP: Hippocampus
pHIP: Posterior hippocampus
aHIP: Anterior hippocampus
PCun: Precuneus
PCun-1: Dorsal-central portion of precuneus
PCun-2: Dorsal-anterior portion of precuneus
PCun-3: Dorsal-posterior portion of precuneus
PCun-4: Ventral portion of precuneus
RSC: Retrosplenial cortex
dlPFC: Dorsolateral prefrontal cortex
AAL: Anatomical automatic labeling
BA: Brodmann’s area
