# Supplementary material for: A specific brain network for a social map in the human brain
Source: Sci Rep. 2022 Feb 2;12:1773. doi: 10.1038/s41598-022-05601-4 (PMC8810806; doi:10.1038/s41598-022-05601-4)
Supplement: Supplementary file 1 — Supplementary Information. [file 41598_2022_5601_MOESM1_ESM.docx]

#### Supplementary Materials

**A specific brain network for a social map in the human brain**

Lu Zhang ^1†^, Ping Chen ^1†^, Matthew Schafer ^2^, Senning Zheng ^1^,

Lixiang Chen ^1^, Shuai Wang ^1^, Qunjun Liang ^1^, Qing Qi ^1^, Yichen Zhang ^1^, Ruiwang Huang ^1^, *

Table S1 shows brain clusters showing significant differences in activation corresponding to the narrative versus optional condition contrast.

Table S2 shows brain clusters showing significant differences in activation corresponding to the narrative and optional condition versus baseline contrast.

Table S3 shows the results of ROI analysis to identify regions that tracking social map information.

Table S4 shows task-dependent functional connectivity obtained from PPI analysis. Here the parametric regressors for vector angle and length were selected as nuisance covariates.

Fig. S1 showed the behaviour test from a participant, which showed how a participant assigned different size of apartments to different characters and put all the characters in a 2D map after finishing the fMRI scanning.

Fig. S2 shows brain clusters with significant activations derived from the GLM analyses.

Fig. S3 shows the locations of the regions of interest (ROI) and the ROIs involved in tracking social map information.

Fig. S4 shows flow chart of the data analysis.

Fig. S5 shows schematic diagram for design matrix of the first-level analysis in GLM2.

**Table S1.** Brain clusters showing significant differences in activation corresponding to the narrative versus optional condition contrast.

| Location of the peak voxel | | Brodmann’s  area (BA) | Peak MNI coordinate | | | Cluster size  (# Voxels) | *t-*value |
| --- | --- | --- | --- | --- | --- | --- | --- |
|  |  |  | *x* | *y* | *z* |  |  |
| ***Optional > narrative*** | | | | | | | |
|  | Thalamus_R |  | 6 | -22 | 0 | 1, 400 | 8.66 |
|  | Parietal_Inf_R | BA40_R | 48 | -52 | 48 | 1, 196 | 8.76 |
|  | Frontal_Inf_Oper_R | BA45_R | 44 | 18 | 6 | 997 | 8.06 |
|  | Frontal_Mid_R |  | 26 | 56 | 36 | 526 | 6.28 |
|  | Frontal_Mid_Orb_R | BA11_R | 30 | 40 | -10 | 440 | 6.35 |
|  | Caudate_R |  | 14 | 14 | 4 | 372 | 6.18 |
|  | Frontal_Mid_R |  | 46 | 32 | 42 | 284 | 5.04 |
|  | Cerebelum_Crus1_R |  | 48 | -48 | -36 | 155 | 5.75 |
|  | Frontal_Sup_R | BA6_R | 30 | -10 | 60 | 111 | 4.65 |
|  | Cingulum_Mid_L | BA32_L | -6 | 26 | 38 | 5, 112 | 9.40 |
|  | Parietal_Inf_L | BA40_L | -48 | -56 | 52 | 2, 145 | 7.56 |
|  | Frontal_Sup_L | BA46_L | -22 | 48 | 24 | 1, 659 | 6.32 |
|  | Insula_L | BA47_L | -34 | 18 | -4 | 1, 587 | 9.08 |
|  | Cingulum_Mid_L | BA23_L | -4 | -18 | 32 | 628 | 6.80 |
|  | Frontal_Mid_Orb_L | BA47_L | -34 | 42 | -10 | 504 | 5.29 |
|  | Caudate_L |  | -12 | 14 | 10 | 406 | 6.59 |
|  | Precuneus_L | BA7_L | -14 | -64 | 52 | 131 | 4.97 |
| ***Narrative > optional*** | | | | | | | |
|  | Temporal_Inf_R | BA20_R | 46 | -22 | -22 | 430 | 6.98 |
|  | Temporal_Pole_Sup_R | BA21_R | 56 | 4 | -12 | 369 | 6.63 |
|  | Fusiform_L | BA20_L | -36 | -12 | -34 | 392 | 5.82 |
|  | Calcarine_L | BA17_L | -4 | -94 | 8 | 14, 603 | 16.6 |
|  | Temporal_Mid_L | BA22_L | -56 | -44 | 10 | 779 | 6.75 |

**Table S2.** Brain clusters showing significant differences in activation corresponding to the narrative and optional condition versus baseline contrast

| Location of the peak voxel | | Brodmann’s  area (BA) | Peak MNI coordinate | | | Cluster size  (# Voxels) | *t-*value |
| --- | --- | --- | --- | --- | --- | --- | --- |
|  |  |  | *x* | *y* | *z* |  |  |
| ***Narrative > baseline*** | | | | | | | |
|  | Fusiform_R | BA19_R | 30 | -76 | -10 | 17, 195 | 15.01 |
|  | Vermis_9 |  | 0 | -52 | -32 | 745 | 13.36 |
|  | Precuneus_R | BA30_R | 8 | -52 | 14 | 660 | 7.37 |
|  | Frontal_Mid_R | BA6_R | 44 | 0 | 54 | 583 | 6.98 |
|  | Temporal_Sup_R | BA22_R | 52 | -6 | -10 | 490 | 7.13 |
|  | Temporal_Sup_R | BA41_R | 50 | -42 | 18 | 288 | 8.20 |
|  | Cerebelum_9_R |  | 20 | -40 | -44 | 206 | 10.23 |
|  | Brain Stem |  | -4 | -32 | -4 | 1, 566 | 23.47 |
|  | Precentral_L | BA6_L | -50 | -2 | 48 | 781 | 7.92 |
|  | Temporal_Mid_L | BA22_L | -54 | -46 | 14 | 474 | 7.59 |
|  | Cerebelum_10_L |  | -22 | -40 | -42 | 282 | 10.42 |
|  | Temporal_Sup_L | BA22_L | -52 | -6 | -10 | 247 | 6.39 |
|  | Fusiform_R | BA19_R | 30 | -76 | -10 | 17, 195 | 15.01 |
| ***Optional > baseline*** | | | | | | | |
|  | Occipital_Inf_R | BA19_R | 42 | -74 | 0 | 6, 500 | 12.76 |
|  | Frontal_Inf_Oper_R | BA48_R | 38 | 8 | 28 | 295 | 6.38 |
|  | Precentral_R | BA6_R | 40 | -2 | 52 | 197 | 5.97 |
|  | Precuneus_R | BA29_R | 8 | -44 | 10 | 192 | 5.52 |
|  | Cerebelum_9_R |  | 20 | -40 | -44 | 123 | 9.06 |
|  | Fusiform_L | BA19_L | -34 | -76 | -12 | 5, 995 | 11.70 |
|  | Brain-Stem |  | -6 | -32 | -4 | 1885 | 16.42 |
|  | Precentral_L | BA6_L | -44 | -2 | 44 | 370 | 5.74 |
|  | Vermis_9 |  | 0 | -54 | -32 | 312 | 9.50 |
|  | Cerebelum_10_L |  | -20 | -40 | -42 | 129 | 8.63 |

**Table S3**. ROI analysis identifying regions that tracking social map information

| Location of the peak voxel | Peak MNI coordinate | | | Cluster size (# Voxels) | *t-*value |
| --- | --- | --- | --- | --- | --- |
|  | *x* | *y* | *z* |  |  |
| *Power>baseline* | | | | | |
| pHIP_L | -20 | -30 | -2 | 30 | 7.27 |
| pHIP_R | 22 | -30 | -2 | 16 | 5.38 |
| PCun_1_L | -10 | -56 | 50 | 141 | 6.45 |
| PCun_2_L | -10 | -54 | 52 | 92 | 5.74 |
| PCun_3_L | -14 | -56 | 16 | 41 | 4.41 |
| PCun_4_L | -10 | -52 | 48 | 23 | 6.02 |
| PCun_1_R | 6 | -56 | 50 | 56 | 5.05 |
| PCun_2_R | 10 | -46 | 48 | 62 | 6.03 |
| PCun_3_R | 12 | -58 | 20 | 126 | 5.92 |
| PCun_4_R | 6 | -50 | 48 | 33 | 5.58 |
| *Affiliation>baseline* | | | | | |
| pHIP_L | -20 | -30 | -2 | 29 | 7.17 |
| PCun_1_L | -6 | -58 | 50 | 160 | 5.70 |
| PCun_2_L | -10 | -50 | 48 | 49 | 5.23 |
| PCun_1_R | 6 | -54 | 50 | 82 | 5.26 |
| PCun_2_R | 10 | -46 | 48 | 51 | 5.70 |
| PCun_3_R | 14 | -58 | 22 | 42 | 4.48 |
| PCun_4_R | 6 | -50 | 48 | 44 | 5.60 |
| *Vector length (V)>baseline* | | | | | |
| pHIP_L | -20 | -30 | -2 | 41 | 11.56 |
| pHIP_R | 18 | -30 | -2 | 41 | 8.74 |
| PCun_3_L | -14 | -62 | 26 | 31 | 5.15 |
| PCun_3_R | 16 | -58 | 24 | 96 | 5.45 |
| *Vector angle (cosθ)>baseline* | | | | | |
| pHIP_L | -20 | -30 | -2 | 28 | 8.20 |
| pHIP_R | 20 | -30 | -2 | 23 | 9.67 |

**Table S4**. Task-dependent functional connectivity obtained from PPI analysis. The parametric regressors for vector angle and length were selected as nuisance covariates

| Location of the peak voxel | | | | Brodmann's  Area (BA) | | Peak MNI coordinate | | | | | Cluster size  (# Voxels) | | | *t-*value | |
| --- | --- | --- | --- | --- | --- | --- | --- | --- | --- | --- | --- | --- | --- | --- | --- |
|  |  |  |  |  |  | *x* | *y* | | *z* | |  |  |  |  |  |
| ***Left posterior hippocampus as ROI*** | | | | | | | | | | | | | | | |
|  | Cuneus_R | | | BA18_R | | 18 | -70 | | 20 | | 159 | | | -5.54 | |
|  | Lingual_L | | | BA18_L | | -10 | -60 | | 2 | | 344 | | | -5.76 | |
|  | Rolandic_Oper_L | | | BA48_L | | -48 | 2 | | 2 | | 139 | | | -5.51 | |
| ***Right posterior hippocampus as ROI*** | | | | | | | | | | | | | | | |
|  | - | | - | | | - | |  | |  |  | |  | |  |
| ***Left precuneus as ROI*** | | | | | | | | | | | | | | | |
|  | Fusiform_R | | | BA37_R | | 36 | -44 | | -18 | | 543 | | | 6.62 | |
|  | Frontal_Inf_Oper_R | | | BA48_R | | 36 | 10 | | 30 | | 303 | | | 6.03 | |
|  | Fusiform_L | | | BA37_L | | -34 | -48 | | -18 | | 208 | | | 6.04 | |
|  | Calcarine_L | | | BA17_L | | -6 | -60 | | 6 | | 261 | | | -5.53 | |
|  | SupraMarginal_L | | | BA42_L | | -60 | -24 | | 16 | | 243 | | | -5.73 | |
| ***Right precuneus as ROI*** | | | | | | | | | | | | | | | |
|  | Fusiform_R | | | BA37_R | | 38 | -58 | | -14 | | 697 | | | 6.97 | |
|  | Frontal_Inf_Oper_R | | | BA48_R | | 38 | 12 | | 24 | | 232 | | | 5.92 | |
|  | Fusiform_L | | | BA37_L | | -32 | -56 | | -14 | | 198 | | | 5.80 | |
|  | Lingual_L | | | BA19_L | | -20 | -64 | | 2 | | 142 | | | -5.16 | |
|  | SupraMarginal_L | | | BA42_L | | -62 | -24 | | 16 | | 133 | | | -5.21 | |

**Table S4** lists the results of the PPI analyses which were robust even though the vector angle and length were included as nuisance covariates in the whole-brain GLM analysis. However, we noticed that the significant connectivity between the right PCun and the insula was affected by the nuisance covariates of the vector angle and length, suggesting that the insula may be involved in tracking social information about the characters' position (vector angle/length) in the social map. According to "global emotional moment" hypothesis (Craig, 2009), during social navigation, we infer that the insula may receive and incorporate internal and external information about characters’ location (vector angle/length) and participants’ emotion. The participants use the information to represent the relationships between themselves and the characters in the outside world at that moment, and to affect decision making.

After fMRI scanning, the participants also finished a behaviour test, which is assigning houses of different sizes to different characters and putting all the characters in a map. Fig. S1 shows an example from one participant:

| 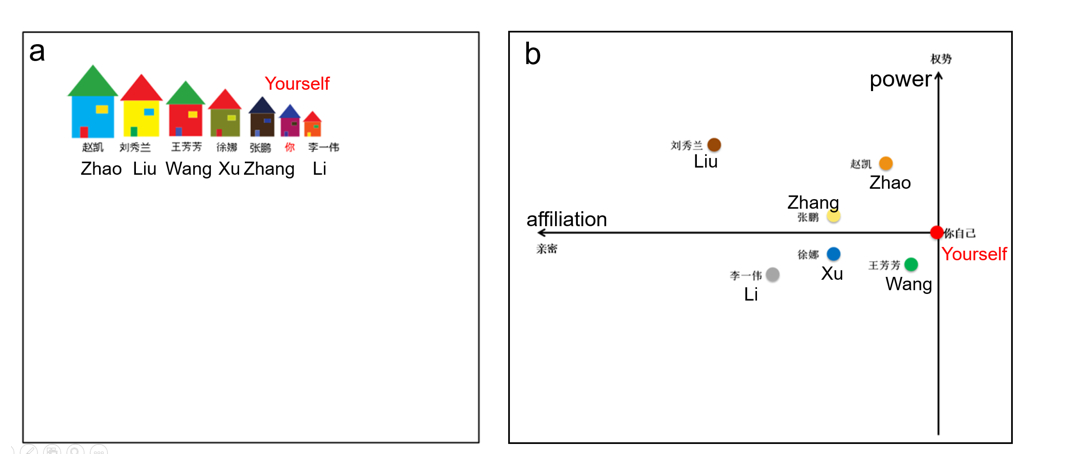 |  |
| --- | --- |

**Fig. S1** (a) Illustration how a participant assigned the apartments to different characters. The sequence is Zhao, Liu, Wang, Xu, Zhang, yourself (the participant), and Li. The character with higher power was assigned a big apartment. Chinese names and the corresponding English names were shown to help understand. (b) Illustration how a participant assigned different characters in the 2D social map. The *x*-axis is affiliation dimension and the *y*-axis is power dimension. Above the *x*-axis, (from left to right) the characters are Liu, Zhang, and Zhao. Below the *x*-axis, (from left to right) the characters are Li, Xu, and Wang. The red dot indicates the participant. Chinese names were translated to the corresponding English names to help understand.

**The story line of task**

In the game, there are six fictitious characters, including five main characters (Miss Wang, Mr. Li, Mr. Zhao, Mrs. Liu, and Miss Xu) and one neutral character (Mr. Zhang) who was a control character. Each participant interacts with all of the six characters. When the story starts, the participant is told that he/she is on a street in the Green City. The 1st character, Wang, approaches the participant and tells the participant that they used to be schoolmates. Wang mysteriously disappears when the 2nd character, Li, shows up. Li behaves as a potential peer. However, the participant later learns that Li has broken up with Wang. From that day forward, there are a lot of communications between the participant and Wang or Li. When the participant is eating with Li in the Zheng Restaurant, the 3rd character, Zhao, a potentially more powerful character, who can help the participant to get a job, is showing up. He mentions knowing the participant’s cousin and creates an affiliation potential, and then hosts a dinner for inviting the participant along with Wang, Li, and Zhang (the 4th character, a control character). The 5th character, Liu, who might directly hire the participant, is well known and admired in the town. The participant is sent for an interview with Liu. Before the interview, the participant meets Liu’s assistant, the 6th character, Xu, at the company. The participant gets the job after a series of interactions with Liu and Xu. The first part of the game ends.

In the second part of the game, the participant knows all of the six characters and keeps interacting with all of them. The participant is working with Liu and Xu and looking for a place to live with the help of all the 6 fictitious characters. The game ends after the last interaction when the participant has to choose whether to rent a flat from Zhao or share an apartment with Xu.


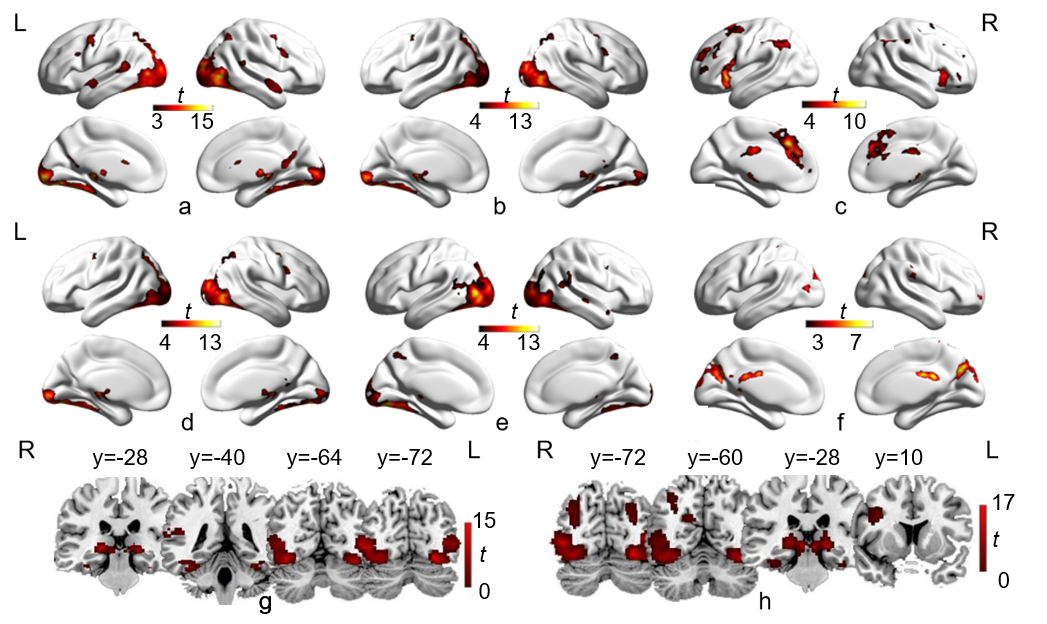


**Fig. S2** Brain clusters with significant activations derived from the GLM analyses. (a) Brain clusters showing significant differences in activation corresponding to the narrative condition versus baseline contrast. (b) Brain clusters showing significant differences in activation corresponding to the optional condition versus baseline contrast. (c) Brain clusters showing significant differences in activation corresponding to the optional condition versus narrative condition contrast. (d) Brain clusters showing significant differences in activation corresponding to the power versus baseline contrast. (e) Brain clusters showing significant differences in activation corresponding to the affiliation versus baseline contrast. (f) Brain clusters showing significant differences in activation corresponding to the power versus affiliation contrast. (g) Brain clusters showing significant differences in activation corresponding to the vector angle versus baseline contrast. (h) Brain clusters showing significant differences in activation corresponding to the vector length versus baseline contrast.

**ROI analysis: subregions of PHG**, **EC, and RSC were not significantly related to power and affiliation**

In addition to the whole brain analysis and the ROI analyses focusing on the hippocampus and the precuneus, we also performed additional ROI analyses in an exploratory way. In most previous studies investigating both spatial and abstract navigation, three regions were gaining increasing attention. They are the parahippocampal gyrus (PHG), EC, and RSCs [1-3]. Do these regions also play different roles in social navigation?

First, we parcellated each of these three subcortical regions into different subregions in standard MNI space to accord with previous studies (Fig. S2a) [3-6] and then analyzed their roles during the role-playing game. Specifically, we parcellated each of the 3 regions (PHG, EC, and RSC) into 4 subregions each in the combined two hemispheres.

The parcellation steps are summarized as follows. (1) We obtained the PHG mask in MNI space from the AAL template and parcellated it into two approximately equal parts along the long axis in each hemisphere. In this way, we obtained the posterior PHG (pPHG) from *y* = 42 to 57 and the anterior PHG (aPHG) from *y* = 57 to 72. (2) We parcellated the EC into two subregions in each hemisphere, the anterior-lateral EC (alEC) and posterior-medial EC (pmEC), following a previous study [5]. (3) We parcellated the RSC into two subregions in each hemisphere, the granular or Brodmann’s Area (BA) 29 and dysgranular areas (BA 30), according to Vann et al. [3].

Second, for each of the ROI, we obtained the parameter estimates images for four contrasts, vector angle-vs-baseline, vector length-vs-baseline, power-vs-baseline, or affiliation-vs-baseline. A one-sample *t*-test was applied in each of the subregions for each of the four contrasts. Gender and age were used as covariates. GRF was used to correct for multiple comparisons. The threshold was set at the voxel level *p* < 0.001 and *p* < 0.05 at the cluster-level. We observed no significant activations in these subregions.


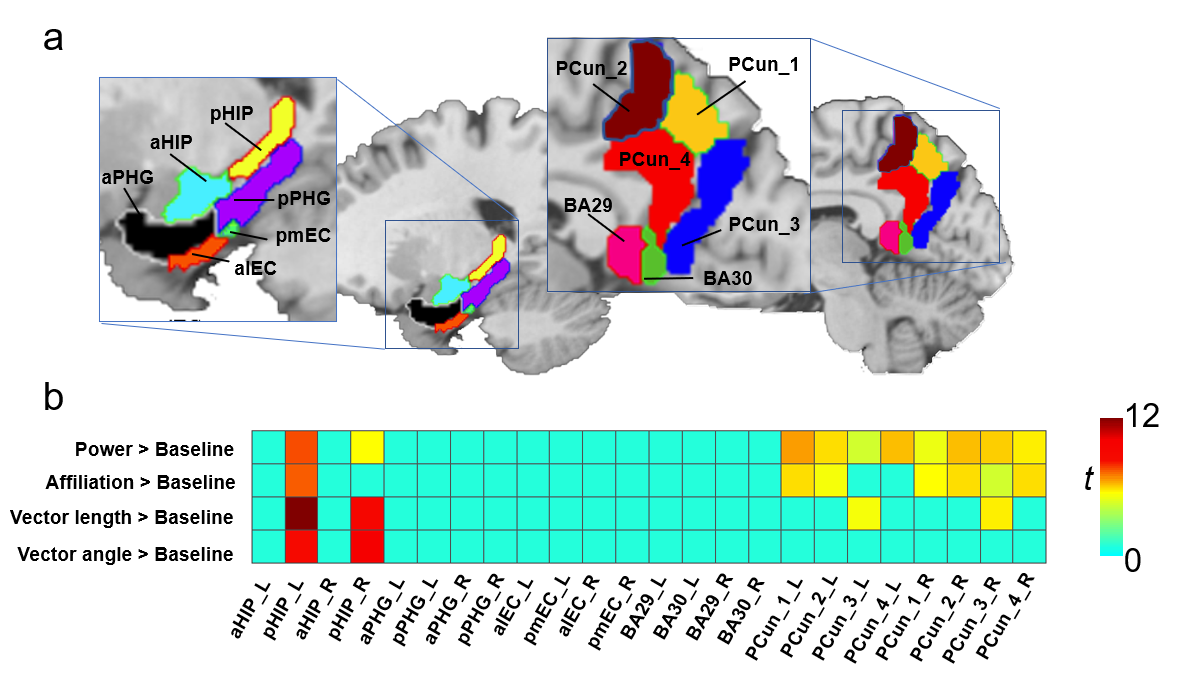
 **Fig. S3** Description of the locations of the regions of interest (ROI) and the ROIs involved in tracking social map information. (a) Colors indicate approximate anatomy distinctions. (b) The brain subregions associated with tracking social map information while the participants were navigating social space. All the results were corrected by GRF at *p* < 0.001 at the voxel level, *p* < 0.05 at the cluster-level. Coordinates in the multicolor box are the location of the peak voxel. Notes: aHIP, anterior hippocampus; pHIP, posterior hippocampus; aPHG, anterior parahippocampal gyrus; pPHG, posterior parahippocampal gyrus; alEC, anterior-lateral entorhinal cortex; pmEC, posterior-medial entorhinal cortex; PCun-1, dorsal-central portion of precuneus; PCun-2, dorsal-anterior portion of precuneus; PCun-3, dorsal-posterior portion of precuneus; PCun-4, ventral portion of precuneus; BA, Brodmann’s Area. Figure S3b is created based on MATLAB R2019B (<https://www.mathworks.com/products/matlab.html>) using the command (matrixplot: <https://www.cxyzjd.com/article/qq_38882446/101272266>).


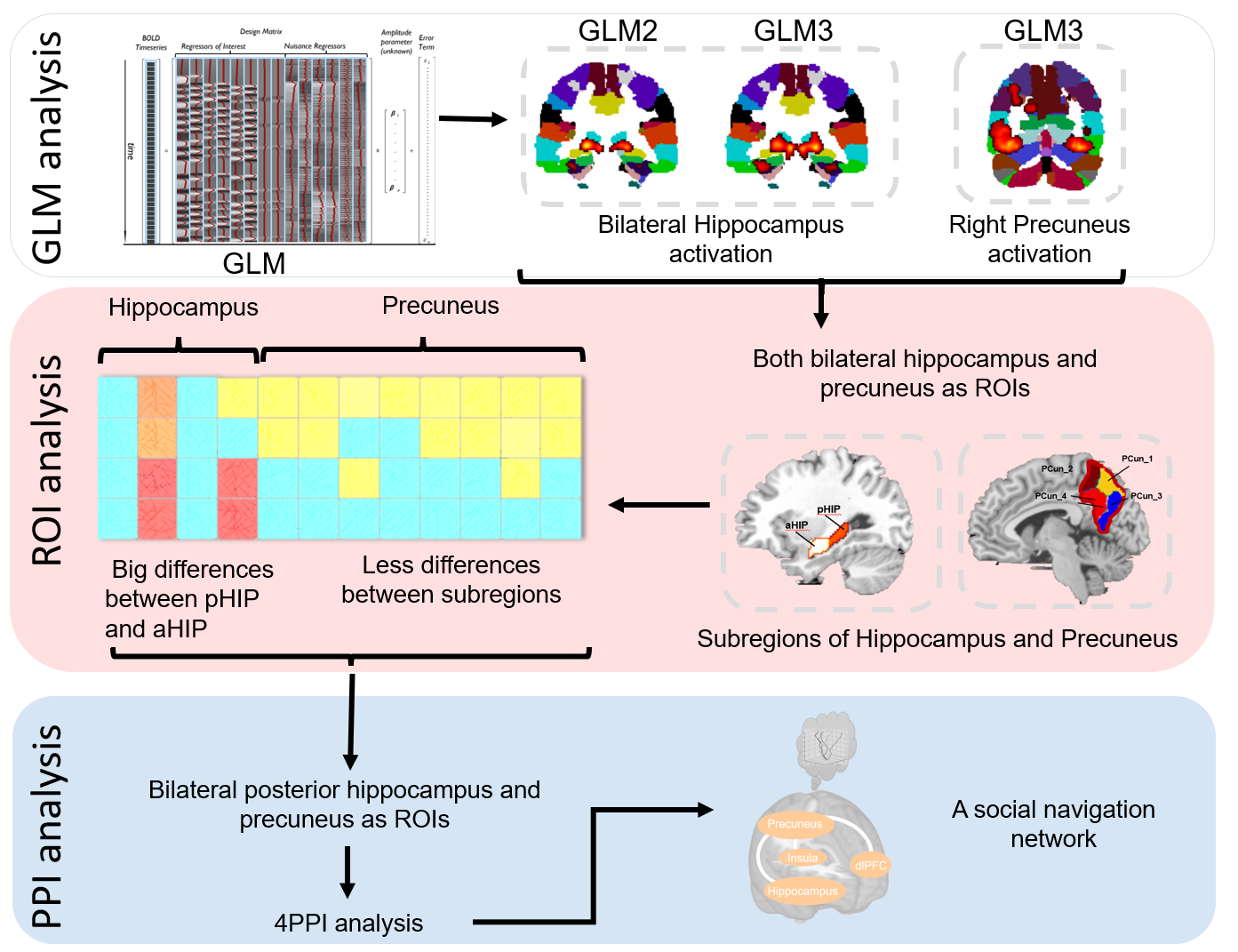


**Fig. S4** Flow chart of the data analysis. GLM2 and GLM3 showed hippocampus activation in bilateral hemispheres, for exploratory purposes, bilateral hippocampus and precuneus were selected to test whether the angle is related to the hippocampus and the length is related to the precuneus for the ROI-based analysis. Specifically, subregions of both hippocampus and precuneus were used as ROIs, respectively, to perform ROI-based analysis. The results suggests that pHIP may play a more important role tracking this type of information in social navigation relative to aHIP while the four subregions of precuneus showed less differences between each other. Thus, we selected four ROIs, including the bilateral pHIP and bilateral precuneus, to perform PPI analysis, respectively, to find a brain network-based model.


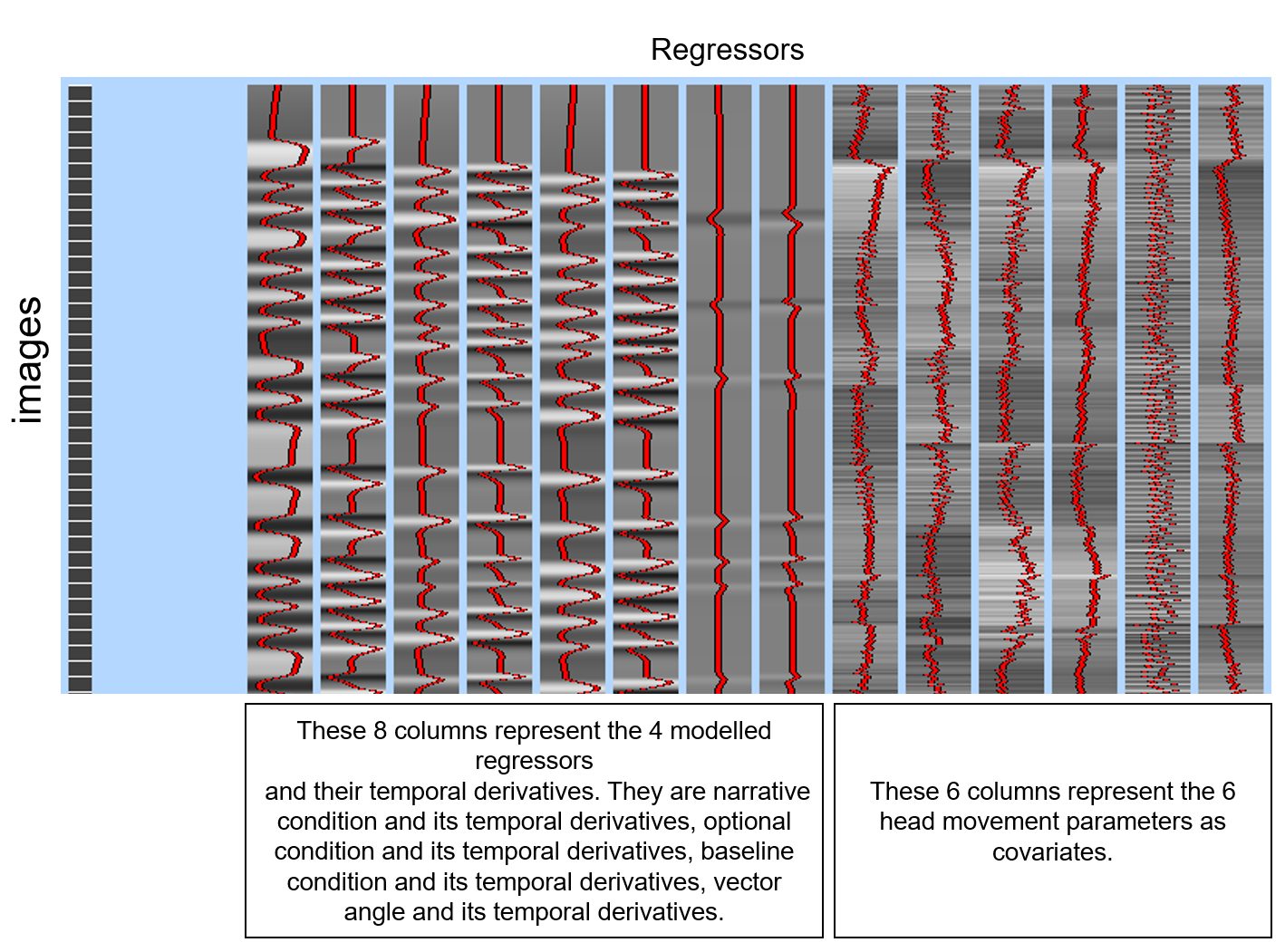


**Fig. S5** Schematic diagram for design matrix of the first-level analysis in GLM2. These left 8 columns represent the 4 modelled regressors and their temporal derivatives. In specific, these columns represent the narrative condition and its temporal derivatives, optional condition and its temporal derivatives, baseline condition and its temporal derivatives, as well as the vector angle (parametric regressor) and its temporal derivatives, respectively. These right 6 columns represent the 6 head movement parameters as covariates.

**References:**

1. Aminoff, E. M., Kveraga, K., & Bar, M. The role of the parahippocampal cortex in cognition. *Trends Cogn Sci.* **17**, 379-390 (2013).
2. Howard, L. R., et al. The hippocampus and entorhinal cortex encode the path and Euclidean distances to goals during navigation. *Curr Biol.* **24**, 1331-1340 (2014).
3. Vann, S. D., Aggleton, J. P., & Maguire, E. A. What does the retrosplenial cortex do? *Nat Rev Neurosci.* **10**, 792-802 (2009).
4. Collin, S. H., Milivojevic, B., & Doeller, C. F. Memory hierarchies map onto the hippocampal long axis in humans. *Nat Neurosci.* **18**, 1562-1564 (2015).
5. Maass, A., Berron, D., Libby, L. A., Ranganath, C., & Duzel, E. Functional subregions of the human entorhinal cortex. *Elife* **4**;10.7554/eLife.06426 (2015).
6. Theves, S., Fernandez, G., & Doeller, C. F. The Hippocampus Encodes Distances in Multidimensional Feature Space. *Curr Biol.* **29**, 1226-1231 (2019).
